# Distinct properties of proteases and nucleases in the gut, salivary gland and saliva of southern green stink bug, *Nezara viridula*

**DOI:** 10.1038/srep27587

**Published:** 2016-06-10

**Authors:** Purushottam R. Lomate, Bryony C. Bonning

**Affiliations:** 1 Department of Entomology, Iowa State University, Ames, 50011, IA, USA

## Abstract

Stink bugs negatively impact numerous plant species of agricultural and horticultural importance. While efforts to develop effective control measures are underway, the unique digestive physiology of these pests presents a significant hurdle for either protein- or nucleotide-based management options. Here we report the comparative biochemical and proteomic characterization of proteases and nucleases from the gut, salivary gland and saliva of the southern green stink bug, *Nezara viridula*. The pH optimum for protease activity was acidic (5 to 6) in the gut with the primary proteases being cysteine proteases, and alkaline (8 to 9) in the saliva and salivary gland with the primary proteases being serine proteases. The serine proteases in saliva differ biochemically from trypsin and chymotrypsin, and the cathepsins in the gut and saliva showed distinct properties in inhibitor assays. Nuclease activity (DNase, RNase, dsRNase) was concentrated in the salivary gland and saliva with negligible activity in the gut. The most abundant proteins of the gut (530) and salivary gland (631) identified by proteomic analysis included four gut proteases along with eight proteases and one nuclease from the salivary gland. Understanding of *N. viridula* digestive physiology will facilitate the design of new strategies for management of this significant pest.

Stink bugs are of significant economic importance to global agriculture. For example, there are more than 50 species of Pentatomid stink bugs impacting 12 major crops worldwide including soybean^1^. A complex of stink bugs, including the southern green stink bug, *Nezara viridula*, the brown stink bug, *Euschistus servus*, the green stink bug, *Acrosternum hilare*, and the brown marmorated stink bug, *Halyomorpha halys* pose particular threats in the US and elsewhere^2^,^3^,^4^. *N. viridula* is a polyphagous insect that feeds on more than 30 families of plants but with a preference for legumes with growing pods^5^. Despite the importance of these pests, fundamental knowledge of stink bug digestive physiology is limited with no comprehensive analysis of digestive enzymes reported.

Stink bugs have piercing-sucking mouthparts, the stylets, that puncture plants causing aesthetic damage. They typically feed on vegetative parts of the plant including leaves, pods and fruits. Some stink bugs such as, *Murgantia histrionica* use a lacerate and flush feeding method, while others, including *N. viridula* use vascular feeding^1^,^2^,^6^. Saliva is released into plant tissues resulting in enzymatic degradation of plant cell components (sugars and lipids), proteins and nucleic acids, and the liquefied digestion products are sucked back for further digestion in the gut^7^,^8^. While release of stink bug saliva and digestive enzymes into the plant to facilitate nutrient extraction is a primary cause of stink bug-associated crop damage^9^,^10^, the biochemical properties of these enzymes are unknown. Insights into stink bug digestive physiology will allow for development of enzymatically stable agents for stink bug control.

Current stink bug management relies exclusively on the use of sprayed, classical chemical insecticides, which are not consistently effective in part due to insecticide resistance^11^,^12^. Understanding of the enzymatic challenges faced by protein- or nucleic acid-based control agents is essential for development of effective new approaches for stink bug control. The focus of the present study was to compare the biochemical properties of digestive proteases and nucleases in the saliva, salivary gland and gut of *N. viridula*, and to identify the most abundant proteases and nucleases in these tissues by proteomic analysis. We established that *N. viridula* uses a two-pronged approach for digestion of proteins with serine proteases predominating in the saliva, and cysteine proteases in the gut. In contrast to the gut, nuclease activities against DNA, RNA and dsRNA were high in the saliva and salivary gland.

## Results

### Salivary gland and gut morphology

**Figure 1:**
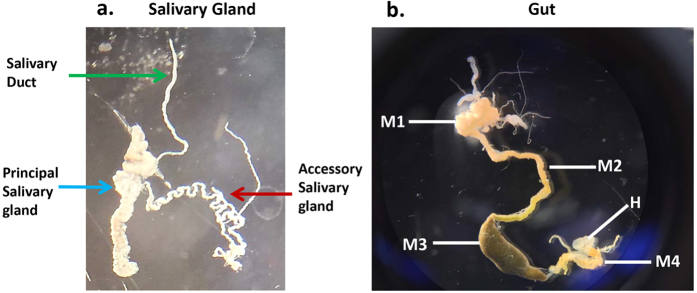
Structure of the salivary gland and gut of *Nezara viridula*. (**a**) Salivary gland: Salivary duct, principal and accessory salivary glands are indicated. The ASG is ~1 cm and the PSG 6–8 mm in length. (**b**) Gut: *N. viridula* gut, with sections according to Tada *et al*.^22^ indicated: M1 first midgut section, M2 second midgut section, M3 third midgut section, M4 fourth midgut section which harbors symbiotic bacteria and does not function in digestion^22^, H hindgut. The length of the entire gut is ~5.5 cm.

*N. viridula* adults have two salivary glands with two major lobes, the principal salivary gland (PSG) and the accessary salivary gland (ASG), along with a salivary duct connected at the junction of the PSG and ASG (Fig. 1). The salivary glands were flanked by and attached to the first section of the midgut. The gut is divided into four sections (M1 to M4) as described by Tada *et al*.^13^ (Fig. 1). Section M4 has a crypt-like structure attached to the hindgut, which harbors symbiotic microorganisms and does not function in digestion^14^.

### Total proteolytic activity and pH optima

**Figure 2:**
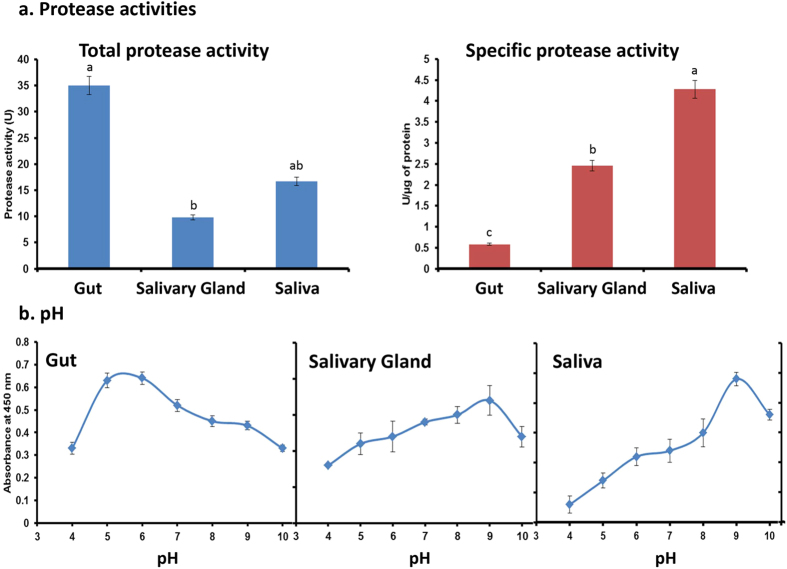
Activity and pH optima for proteases in the gut, salivary gland and saliva of *Nezara viridula*. (**a**) Total protease and specific protease activities: Azocasein was used as a general protease substrate. (**b**) pH optima for total protease activities: Total protease activity was assayed in different buffer systems from pH 4.0 to 10.0 using azocasein as substrate. Error bars indicate standard deviation. Treatments with different letters are significantly different (*P* ≤ 0.01; Student’s t-test).

The total protease activity and specific protease activity for *N. viridula* gut, salivary gland and saliva using azocasein as substrate are shown in Fig. 2. While the total protease activity was highest in the gut, the specific activity was lowest in the gut compared to saliva and salivary gland (Fig. 2). The high specific protease activity in saliva reflects the relatively low protein content. The optimum pH for protease activity in the salivary gland and saliva was 8 to 9 and in the gut was 5 to 6 (Fig. 2).

### Inhibitor assays

**Figure 3:**
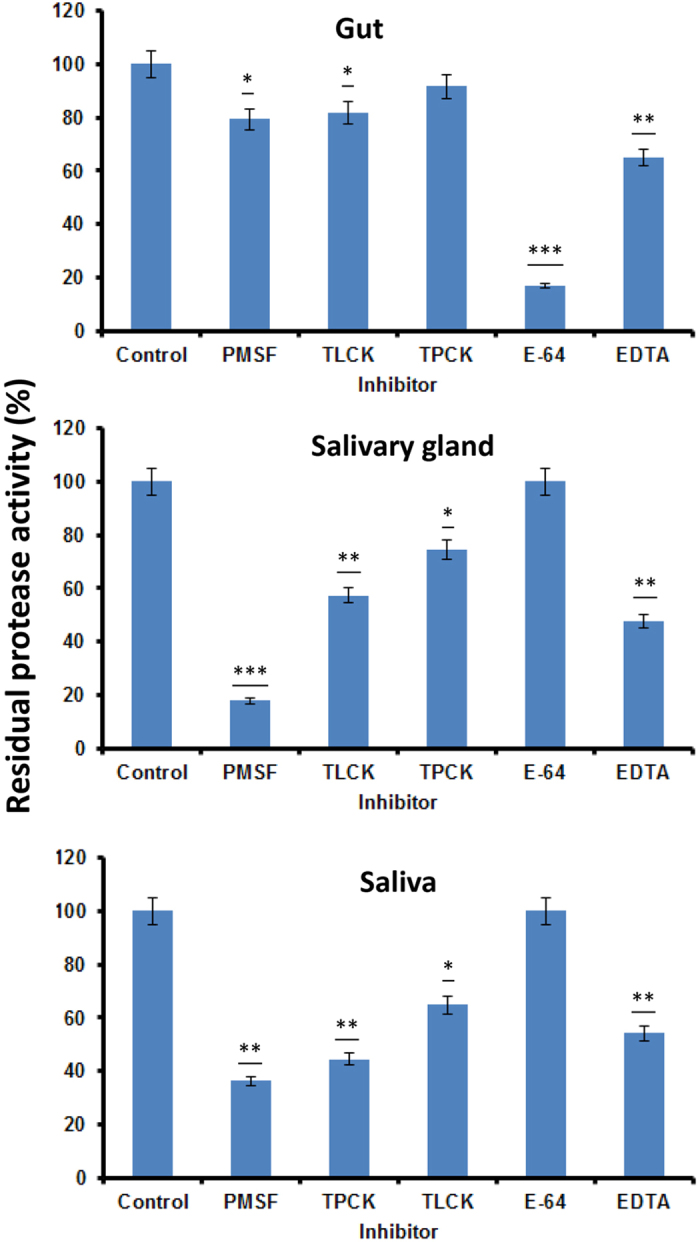
Impact of synthetic protease inhibitors on total protease activity in gut, salivary gland and saliva of *Nezara viridula*. Samples were mixed with inhibitors and pre-incubated for 30 min at 37 °C. Residual protease activity was assayed through spectrophotometric assay using Azocasein as substrate. Protease activity without inhibitor (control) was considered 100%. Statistical comparisons were conducted for each treatment compared to the control (*P ≤ 0.01; **P ≤ 0.001; ***P ≤ 0.0001; Student’s *t*-test).

The profile of protease inhibition differed significantly between the gut and salivary gland or saliva (Fig. 3). The cysteine protease inhibitor E-64 inhibited more than 90% of gut protease activity but did not affect protease activity from saliva or salivary gland. However, protease activity in the salivary gland and saliva was significantly inhibited (~80% inhibition) by a class-specific serine protease inhibitor PMSF. The trypsin inhibitor TLCK inhibited 20%, 80% and 70% of protease activity from the gut, salivary gland and saliva respectively. The chymotrypsin inhibitor TPCK inhibited around 40% of protease activity from saliva or salivary gland, but only 10% activity from gut (Fig. 3). These results indicate that cysteine proteases predominate in the gut, with trace amounts of trypsin-like and chymotrypsin-like activity. In contrast, serine proteases predominate in the saliva and salivary gland, along with significant metalloprotease activity.

### Specific substrate and class specific inhibitor assays

**Figure 4:**
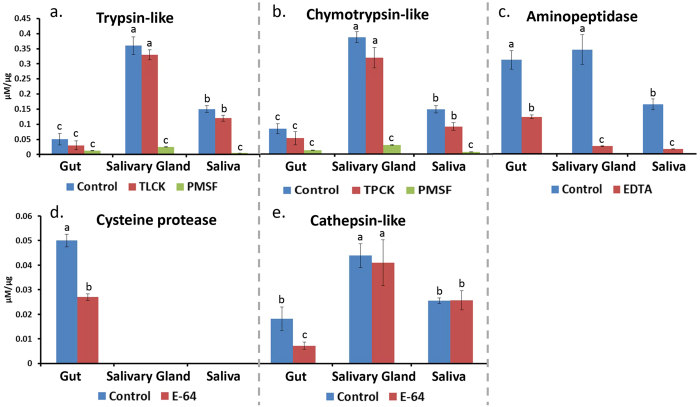
Activities of specific protease types in different tissues of *Nezara viridula* as shown by the use of class-specific inhibitors. Samples mixed with inhibitors were pre-incubated for 30 min at 37 °C. Residual protease activity was assayed using specific chromogenic substrates such as (**a**) BApNa for trypsin, (**b**) SAAPFpNA for chymotrypsin, (**c**) LpNA for aminopeptidase, (**d**) pGPLpNA for cysteine protease, and (**e**) AApNA for cathepsin B. Statistical differences between treatments for each enzyme type are indicated with different letters indicating significant differences between groups (*P* ≤ 0.05; Student’s *t*-test).

To delineate the specific types of protease activity present, different chromogenic substrates were used with a combination of inhibitors which are specific to a selective protease. All five enzyme types were detected in the gut (Fig. 4). Salivary gland and saliva had significantly higher chymotrypsin-like, trypsin-like and cathepsin activities compared to the gut (*P* ≤ 0.05; Student’s *t*-test), and no cysteine protease activity. In contrast, cysteine protease activities were the most abundant in the gut compared to saliva and salivary gland. PMSF significantly inhibited protease activity from salivary gland and saliva, but TLCK and TPCK did not significantly inhibit trypsin and chymotrypsin activity (Fig. 4). These results indicate the presence of serine proteases with different specificity (other than trypsin and chymotrypsin) in salivary gland and saliva. As expected, E-64 significantly inhibited gut cysteine protease activity, and EDTA inhibited aminopeptidase activity from gut, saliva and salivary gland. While E-64 inhibited ~50% gut cathepsin, the same activity was not inhibited by E-64 in salivary gland and saliva (Fig. 4). This result indicates that cathepsins in the gut are cysteine proteases (sensitive to E64) while cathepsins in saliva belong to a different cathepsin class. Taken together, high cysteine protease activity was found in the *N. viridula* gut, while high serine protease, cathepsin and aminopeptidase activities were found in salivary gland and saliva.

### Nuclease activities

**Figure 5:**
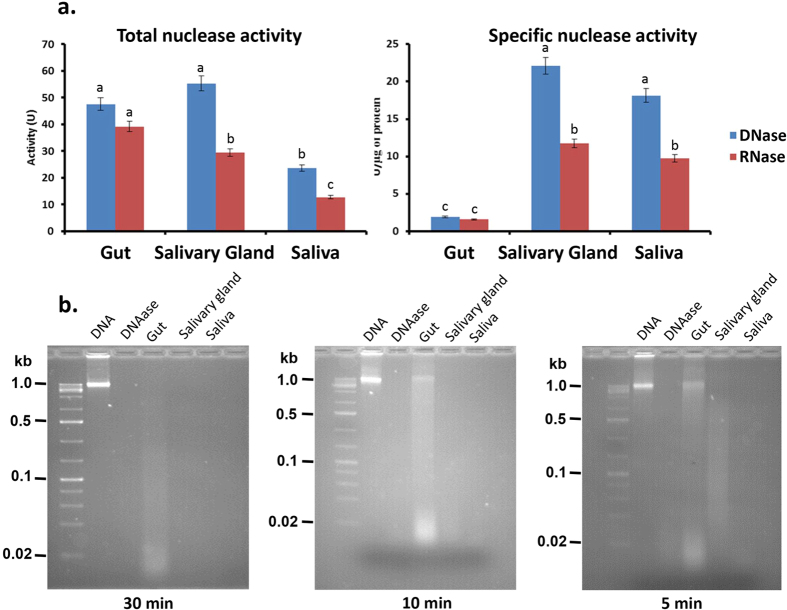
DNase and RNase activities and degradation of DNA by *Nezara viridula* nucleases. (**a**) Total and specific nuclease activities in gut, salivary gland and saliva (letters indicate significantly different groups; *P* ≤ 0.05; Student’s *t*-test). (**b**) Degradation of DNA by DNases from gut, salivary gland and saliva. Samples (gut, salivary gland or saliva) were incubated with DNA for 5, 10 and 30 min. Samples were then run on 2% agarose gels and DNA visualized by ethidium bromide staining. TURBO™ DNase (DNase) was used as a positive control.

**Figure 6:**
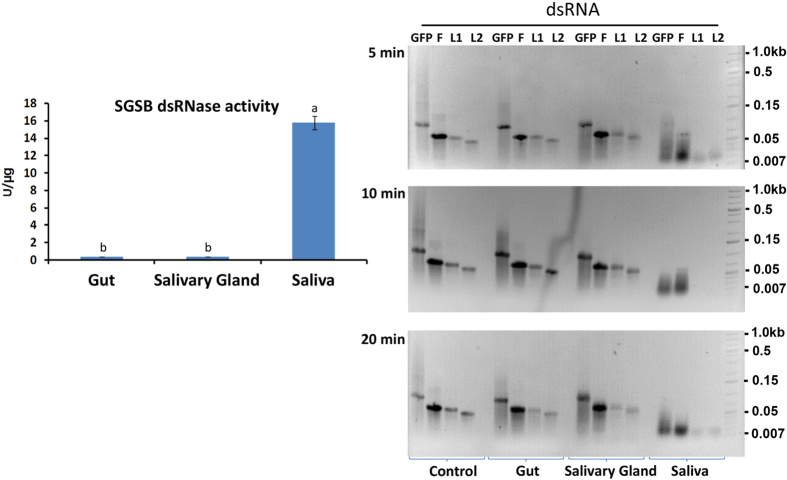
Degradation of dsRNA by *Nezara viridula* gut, salivary gland and saliva. The dsRNase assay was performed using non-relevant dsRNA (cathepsin F dsRNA from *Brugia malayi*) as substrate. Different letters indicate significant differences between groups (*P* ≤ 0.05; Student’s *t*-test). Agarose gels at right show dsRNA degradation patterns. For this analysis samples (gut, salivary gland, saliva) were incubated with dsRNAs (*B. malayi* cathepsin isoforms F, L1, L2 and green fluorescent protein, GFP, dsRNAs, as indicated) for 5, 10 and 20 min. Samples were then run on 2% agarose gels and dsRNA degradation profiles visualized by ethidium bromide staining of the gel.

The DNase, RNase and dsRNase activities in *N. viridula* gut, salivary gland and saliva were determined. For DNase and RNase activities, the specific activities were highest in saliva and salivary gland, and relatively low in the gut (Fig. 5a). The degradation pattern of DNA visualized on agarose gels supported the relative levels of nuclease activity with complete degradation of DNA by salivary gland and salivary enzymes within 5 min, and relatively little degradation by gut enzymes (Fig. 5b). The specific activity of dsRNase was the highest in saliva at 15.76 U/μg with only low activity (0.385 U/μg) in gut and salivary gland samples (Fig. 6). The rate of dsRNA degradation visualized in agarose gels reflected the activity assay data with only minimal dsRNA degradation seen in gut and salivary gland samples at 20 min (Fig. 6). Taken together, these results demonstrate that nucleases (DNase, RNase, dsRNase) are abundant in *N. viridula* saliva and salivary gland, but present at relatively low levels in the gut.

### Proteomic identification of proteases and nucleases

**Figure 7:**
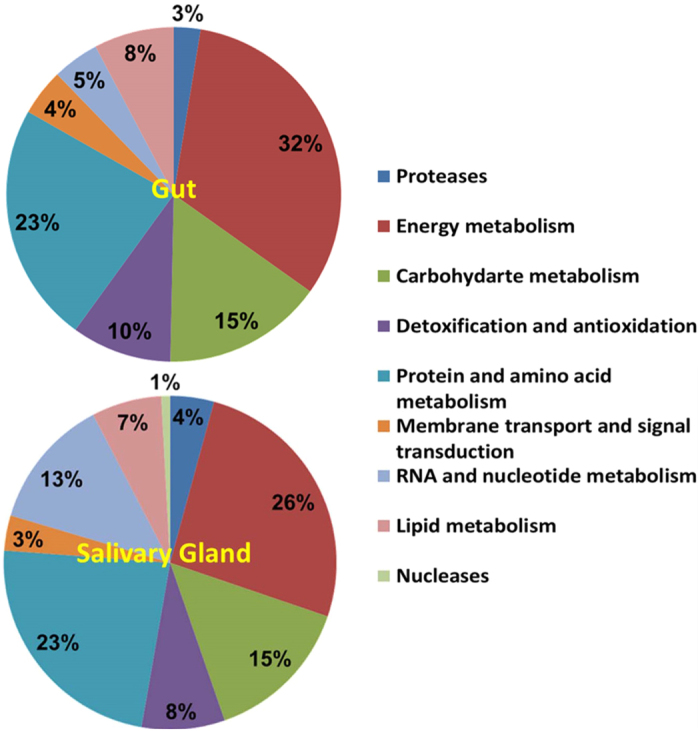
Functional annotation of enzymes identified from the *Nezara viridula* gut and salivary gland proteomes. Enzymes identified (highlighted in Supplementary File 1) were functionally annotated and classified according to metabolic class. Data are shown for comparison of enzyme function in the gut and salivary gland proteomes.

Proteomic analysis to identify proteases and nucleases from *N. viridula* resulted in identification of 530 and 631 proteins from the gut and salivary gland respectively (Supplementary File 1). Identified enzymes (highlighted in Supplementary File 1) were computationally annotated on the basis of their metabolic function. Approximately 32% of gut and 26% of the salivary gland enzymes were involved in energy metabolism whereas 23% of the enzymes function in protein metabolism (Fig. 7). Significant proportions of the enzymes identified function in detoxification and antioxidation (10% gut and 8% salivary gland), and in carbohydrate metabolism (15%). Proteases comprised 3% and 4.25% of enzymes from the gut and salivary gland respectively (Fig. 7). The higher number of proteases and nucleases detected in the salivary gland compared to the gut supports the higher activity levels for some protease and nuclease groups (e.g. serine proteases and dsRNases) in salivary gland extracts.

Four gut proteases were identified along with eight proteases and one nuclease from salivary gland. The gut proteases were one aminopeptidase, two putative aminopeptidases and one cathepsin-like cysteine proteinase (Table 1). In the salivary gland, aminopeptidases and metalloproteases with cathepsin D, cysteine protease and a nuclease were identified (Table 1). Sequence alignment showed that although the gut and salivary gland proteases belong to similar mechanistic classes, their sequences differ (Supplementary Fig. S1). An exception was aminopeptidase N-10 which shared 100% sequence identity between the two tissues. Salivary gland proteases, including two metalloproteases, one methionine aminopeptidase and a ubiquitin-specific peptidase formed a separate clade on phylogenetic analysis. A cathepsin L-like cysteine protease from the gut and a “26,29 kDa proteinase-like” cysteine protease from salivary gland shared ~30% sequence identity and formed a separate group. An aspartate protease cathepsin D and a signal peptidase from salivary gland shared very low sequence similarity with other proteases and stand alone in the phylogenetic tree (Supplementary Fig. S1). Both sequence analysis and activity assays highlight the presence of different proteases and nucleases in the gut and salivary gland.

## Discussion

The use by stink bugs of both extra-oral and gut-based digestion of host plant materials using enzymes with distinct pH optima allows for a two-pronged approach for efficient use of nutrient value from plant proteins, and for efficient degradation of toxins or plant defense molecules. The high enzymatic activity for efficient digestion likely reflects the unusual digestive physiology of these insects, with section M4 of the alimentary tract harboring symbiotic bacteria and being blocked to ingested material^14^, requiring complete digestion and absorption of nutrients in sections M1-M3. The enzymatic challenge faced by material ingested by stink bugs poses a significant problem however for development of novel strategies for stink bug management. Insect specific toxins such as those derived from the bacterium *Bacillus thuringiensis*^15^, and silencing RNAs designed to block translation of insect proteins essential for survival^16^,^17^, are likely to be inactivated before reaching their target site within the stink bug. Here we undertook biochemical characterization of digestive enzymes in the gut, salivary gland and saliva of *N. viridula* to define the overall enzymatic composition of these tissues.

The structure of the *N. viridula* salivary gland was comparable to that of the brown marmorated stink bug, *Halyomorpha halys*^18^, with two lobes, the accessary and principal salivary glands that produce watery and sheath (or gelling) saliva respectively^19^,^20^. Consistent with high enzymatic activity in watery saliva of other species^21^, *N. viridula* saliva had the highest specific protease activity of the three tissues. Based on the alkaline pH optimum, and sensitivity to serine protease inhibitors, the salivary proteases of *N. viridula* are predominantly of the serine mechanistic class. Interestingly, protease activity in salivary gland and saliva was inhibited by PMSF, a class-specific serine protease inhibitor, while the activity was not inhibited by TLCK and TPCK, specific inhibitors of trypsin and chymotrypsin-like enzymes respectively. *N. viridula* therefore has serine proteases in saliva that differ from trypsin and chymotrypsin. Similarly, the gut and salivary cathepsins differ as gut cathepsins were inhibited by E-64 (a cysteine proteases inhibitor) but salivary gland and salivary cathepsins were not.

While the biochemical characteristics of stink bug digestive enzymes have not previously been assessed, the digestive enzymes of two other species of Hemiptera, both predaceous insects in the suborder Heteroptera have been investigated. The salivary and gut proteases of *Zelus renardii* (Reduviidae)^22^,^23^ showed optimal activity at pH 7 to 8.5, with serine proteases predominant in the salivary gland, and carboxypeptidase and aminopeptidase in the gut. In contrast to *N. viridula*, which has aminopeptidase activity in both the gut and saliva, aminopeptidase activity was only detected in the gut of *Z. renardii*. Cysteine protease activity was not assessed. The pH optima and enzyme distribution in the spined soldier bug, *Podisus maculiventris* (Pentatomidae)^24^ was more comparable to that of *N. viridula* (Pentatomidae) including differential sensitivity of gut and salivary proteases to different inhibitors.

Similar to the gut environment of Coleoptera and other Hemiptera, and in contrast to that of Lepidoptera^25^, an *N. viridula* gut pH of 5.5 was recorded with cathepsins as the predominant protease type. These results are consistent with the pH optima and enzymatic composition in the gut of other Hemiptera, such as the predatory bug *Podisus maculiventris*, *Rhodnius prolixus* and *Euschistus euschistoides*^23^,^24^. Trace amounts of trypsin and chymotrypsin were also found in the gut of *P. maculiventris*^26^, similar to *N. viridula*. Knowledge of the proteolytic composition of the *N. viridula* saliva and gut lumen environments provides for direct suppression of proteases through plant expression of protease inhibitors^27^,^28^. The application of protease inhibitors to disrupt digestive processes for suppression of lepidopteran pests however resulted in upregulation of other protease types, highlighting plasticity within insect digestive physiology^28^. Similar adjustment may result from exposure of stink bugs to plant-expressed protease inhibitors.

While dsRNase activity has been detected in the gut of insects such as the lepidopteran, *Bombyx mori* and orthopteran, *Schistocerca gregaria*^29^,^30^,^31^, the release of salivary ribonucleases by Hemiptera into host plant tissues represents a particular problem for application of RNA interference-based approaches for suppression of hemipteran pests. Saliva and salivary gland extract from the tarnished plant bug *Lygus lineolaris* rapidly degraded dsRNA^32^, dsRNase was released during feeding of the pea aphid, *Acyrthosiphon pisum*^33^ and high dsRNase activity levels were detected in the saliva of *N. viridula* in the present study. The high level of dsRNase activity in saliva compared to the very low level of activity detected in the salivary gland suggests that dsRNases are produced as zymogens that are activated on release into the saliva. This in turn suggests that the presence of active dsRNases in the salivary gland may be harmful to the stink bug. In addition to dsRNase activity, high levels of specific DNase and RNase activity were detected in the salivary gland and saliva of *N. viridula*. The DNase activity levels were comparable to that of the purified commercial enzyme (TURBO™ DNase), suggesting an important role for DNases in the digestive process and / or defense. Nucleases in saliva directly or indirectly protect insects from infection by viruses present in the host plant, be they insect pathogens, or insect vectored viruses of plants^34^. Nucleases therefore are likely to provide defense against pathogens, in addition to mediating digestion of host plant-derived nucleic acids.

The results of functional annotation of enzymes identified by proteomic analysis were consistent with the roles of the gut and saliva in digestion, detoxification, and protection against microbes. Digestive enzymes comprised 23% of identified proteins, consistent with proteomic analysis of *H. halys* watery saliva in which 29% of identified proteins were digestive enzymes^18^. The abundance of proteases and nucleases in the gut and salivary gland of *N. viridula* is similar to enzyme abundance in other insect species^35^,^36^, and the divergence in sequences between salivary and gut proteases supports the presence of enzymes with distinct properties in these two tissues. The abundance of aminopeptidases in the gut and salivary gland proteomes was in accordance with high aminopeptidase activity in both tissues.

Our results highlight the different enzymatic composition of *N. viridula* salivary gland and gut for efficient digestion of host plant components, detoxification of proteins used in plant defense responses, and for defense against microbes such as viruses. The distinct profiles of protease and nuclease activities in the two tissues present a unique challenge for the design of control agents that can withstand such a two-pronged enzymatic attack.

## Methods

### Chemicals

Azocasein, phenyl methyl-sulfonyl fluoride (PMSF), tosyl phenylalanyl chloromethyl ketone (TPCK), tosyl lysine chloromethylketone (TLCK), E-64 (10 μM), ethylenediaminetetraacetic acid (EDTA), N-α-benzoyl-Arg p-nitroanilide (BApNA), N-succinyl-Ala-Ala-Pro-Phe p-nitroanilide (SAAPFpNA), leucine p-nitroanilide (LpNA), p-Glu-Phe-Leu-pNA (pGPLpNA), Z-Arg-Arg-pNA (AApNA), calf thymus DNA and baker’s yeast RNA were purchased from Sigma-Aldrich, St. Louis, MO, (USA).

### Colony maintenance, dissection and collection of saliva

*N. viridula* were reared under controlled conditions (16 h light; 8 h dark, 27–28 °C, 65% humidity) and fed a mixed diet containing green beans, sweet corn, carrots and raw peanuts. Adults were chilled for one hour over ice to render them immobile and then dissected. For enzyme assays, the midgut and salivary glands were isolated under a dissection microscope in a plastic Petri dish filled with phosphate-buffered saline (PBS: 0.1 M pH 7.0). Isolated tissues were snap frozen in liquid nitrogen and kept at −80 °C until use. Saliva from *N. viridula* adults was collected as described previously^13^. Briefly, adult insects were chilled on ice for five min, then placed in a Petri dish, ventral side up and observed under a dissecting microscope. As the insects warm to room temperature, watery saliva is released from the tip of the beak. This saliva was collected into filter pipet tips and expelled into a 1.5 ml tube containing 10 μL of phosphate-buffered saline (0.1 M pH 7.0). Samples were stored at −80 °C until use.

### Preparation of gut and salivary gland extracts

Whole guts and salivary glands were ground in liquid nitrogen to a fine powder using a mortar and pestle. The tissue powder was homogenized in phosphate-buffered saline (0.1 M pH 7.0) at a ratio of 1:6 (w/v) and the homogenate was centrifuged twice at 10,000 g at 4 °C for 20 min. The supernatant was collected and used as gut or salivary gland extract. Protein concentrations in the extracts were determined by the Bradford method^37^ using BSA as standard.

### Total proteolytic activity assay

Total proteolytic activity in gut, salivary gland and saliva (5 μg of protein from each sample) was determined using azocasein as substrate^38^ with 1% (w/v) solution of the substrate in assay buffer (0.1 M Tris-HCl pH 7.0). Gut, salivary gland extracts and saliva (10 μL) were added to 200 μL of substrate and incubated overnight at 37 °C. The reaction was terminated by adding 300 μL of 5% trichloroacetic acid (TCA). Precipitated proteins were centrifuged (10,000 g, 10 min) and 0.5 mL of the supernatant was added to 0.5 mL of 1 M sodium hydroxide. The absorbance was measured at 450 nm (Nanodrop 2000 spectrophotometer; Thermo Fisher Scientific, Waltham, MA, USA) and an increase of 0.01 OD per min defined as one protease activity unit.

### Optimum pH of proteolytic activity

The pH optima of *N. viridula* proteolytic activities in different tissues were determined by adding extracts (10 μL each) to buffers (100 μL) with pH ranging from 4 to 10 (pH 4 to 10 buffers; sodium- acetate pH 4, 5 phosphate pH 6, 7 Tris-HCl pH 8 and glycine-NaOH pH 9, 10) added to 100 μL of azocasein prepared in distilled water. The assays were carried out as described above.

### Protease inhibition assays

The synthetic inhibitors PMSF (5 mM), TPCK (100 μM), TLCK (100 μM), E-64 (10 μM) and EDTA (10 μM), were mixed with gut extract, salivary gland extract and saliva samples (5 μg protein per sample in a volume of 10 μL) and incubated for 30 min at 37 °C. The protease extract-inhibitor mixture was added to azocasein solution (200 μL, prepared in phosphate buffer 0.1 M pH 6.0 for gut and Tris-HCl buffer 0.1 M pH 8.5 for salivary gland and saliva). Reactions were incubated at 37 °C and terminated by addition of TCA (300 μL, 5%). Protease activity was determined as described above.

### Specific substrate and class-specific inhibitor assays

The specific substrates and inhibitor combinations used were 1) BApNA and TLCK or PMSF, 2) SAAPFpNA and TPCK or PMSF, 3) LpNA and EDTA, 4) pGPLpNA and E-64, and 5) AApNA and E-64. Substrates were solubilized in DMSO prior to dilution in buffer (phosphate buffer pH 6.0 for gut, Tris-HCl pH 8.5 for salivary gland and saliva). Synthetic inhibitors listed above were mixed with the gut and salivary gland extract, and saliva samples (10 μL each) and incubated for 30 min at 37 °C. Reaction mixtures (20 μL) were added to 100 μL of each substrate (10 mM) and incubated overnight at 37 °C. Reactions were terminated by adding acetic acid (100 μL, 30%). The absorbance was measured at 410 nm and protease activity defined as production of 1 μmol of p-nitroaniline/mg of protein/min at 37 °C.

### Nuclease activity assays

DNase, RNase and dsRNase activities were determined on the basis of the release of acid soluble hydrolysis products from DNA, RNA or dsRNA^39^. Calf thymus DNA or yeast RNA was used as substrate, dissolved in 20 mM Tris-HCl, pH 8.0 containing 25 mM NaCl, 10 mM MgCl2, and 5 mM CaCl2. Gut, salivary gland extracts and saliva (5 μg protein per sample in a volume of 10 μL) were added to 200 μL DNA or RNA (100 μg/mL) and the reaction was carried out at 37 °C for 30 min. TURBO™ DNase (5 units) (Life Technologies, Carlsbad, CA, USA) and RNase A (20 μg) (Thermo Scientific, Waltham, MA, USA) were used as positive controls. The reaction was stopped by addition of 300 μL of 10% TCA containing 20 mM sodium pyrophosphate. For dsRNase activity assays, dsRNAs were prepared using the Ambion^®^ MEGAscript^®^ RNAi Kit (Life Technologies, Carlsbad, CA, USA) following the manufacturer’s protocol. A non-relevant dsRNA (*Brugia malayi* cathepsin F gene sequence) was used as substrate. Samples (10 μL) were added to 20 μL substrate (0.1 μg/μL in buffer as described above) and the reaction was carried out at 37 °C for 30 min. The reaction was stopped by the addition of 30 μL of 10% TCA containing 20 mM sodium pyrophosphate. The nucleic acids from all reactions were precipitated on ice for 1 h and centrifuged (10,000 g, 10 min). Absorbance of the supernatant was measured and one unit of enzyme activity defined as an increase in *A*_260_ of 0.01 per min.

### Degradation of nucleic acids

Calf thymus DNA (1 μg/μL) was incubated with gut, salivary gland extract and saliva (2 μg protein per sample in a volume of 5 μL) at 37 °C for 5, 10, or 30 min. Samples were run and visualized on agarose gels (2%). *N. viridula* nuclease activity was assayed using GFP dsRNA and non-relevant dsRNA (*Brugia malayi* cathepsin F, L1 and L2). dsRNAs (2 μL each) were incubated with tissue samples (5 μL each) for 5, 10, or 20 min and degradation assessed following agarose gel electrophoresis. Nucleic acids in the gel were visualized using ethidium bromide.

### Proteomics

Gut (midgut and hindgut) and salivary gland (ASG, PSG and salivary duct) tissues were isolated from adult *N. viridula*, snap frozen, and stored at −80 °C. Tissues were ground in liquid nitrogen and mixed with 1mL Trizol in a vial containing glass beads. The mixture was homogenized using FastPrep®-24 homogenizer (MP24, MP Biomedicals, Solon, OH, USA) and centrifuged (10,000 g/20min/4 °C). Proteins from the supernatant were isolated using the Invitrogen Protein Isolation Kit (Invitrogen, Carlsbad, CA, USA). Supernatant and pellet samples were mixed with Agilent Isoelectric focusing (IEF) OFFGEL buffer, separated by SDS-PAGE (12% gel) and visualized by silver staining (Supplementary Fig. S2). Protein bands were excised from the gel and in gel digested with trypsin according to published procedures^40^. Digested peptides were analyzed by LC-MS/MS. All MS/MS samples were analyzed using Mascot (Matrix Science, London, UK; version 2.5.1). Proteins were identified with reference to the Hemipteran Database and to the *H. halys* translated transcriptome^41^. Mascot was set up to search the *H. halys* translated transcriptome_20150105 database (4794 entries) and the hemipteran protein database downloaded from Swiss-Prot (TrEMBL_LarvalBugs_20141201 98776 entries) assuming digestion with trypsin. Mascot was searched with a fragment ion mass tolerance of 0.60 Da and a parent ion tolerance of 0.75 Da. Carbamidomethyl of cysteine was specified in Mascot as a fixed modification.

### Criteria for protein identification

Scaffold (version Scaffold_4.4.1.1, Proteome Software Inc., Portland, OR, USA) was used to validate MS/MS based peptide and protein identifications. Peptide identifications were accepted if they could be established at >95.0% probability by the Peptide Prophet algorithm^42^ with Scaffold delta-mass correction. Protein identifications were accepted if they could be established at >99.0% probability and contained at least 3 identified peptides. Protein probabilities were assigned by the Protein Prophet algorithm^43^. Proteins that contained similar peptides and could not be differentiated based on MS/MS analysis alone were grouped to satisfy the principles of parsimony. Proteins with enzymatic activity were functionally annotated with GO terms from gene_association.goa_uniprot (downloaded Nov 21, 2015)^44^. For phylogenetic analyses, protease sequences representing the peptides identified in gut and salivary gland were extracted from the database (BMSB translated transcriptome_ 20150105 and TrEMBL_LarvalBugs_20141201 database) that were used for proteomic identification. The sequence analysis was performed using EMBL-EBI Multiple Sequence Alignment Tool, ClustalW2 (http://www.ebi.ac.uk/Tools/msa/clustalw2/) and phylogenetic tree was generated.

### Statistical Analysis

All experiments with the exception of proteomic analysis were performed with two biological replicates, each with three technical replicates and with each sample assayed twice. The Student t-test was used to assess statistical significance for enzyme activity assays.

## Additional Information

**How to cite this article**: Lomate, P. R. and Bonning, B. C. Distinct properties of proteases and nucleases in the gut, salivary gland and saliva of southern green stink bug, *Nezara viridula*. *Sci. Rep.* **6**, 27587; doi: 10.1038/srep27587 (2016).

## Supplementary Material

Supplementary File 1

Supplementary File 2

## Figures and Tables

**Table 1:** Proteases and nucleases identified in the gut and salivary gland of *Nezara viridula* by proteomic analysis.

Accession No	Protein name	Insect species	Mr (kDa)	Sequence Coverage (%)	Protein Identification Probability
**Proteases (Gut)**					
AAO48766	Cathepsin L-like cysteine proteinase	*Tenebrio molitor*	34	3.3	100%
AFK85026	Aminopeptidase N-10	*Bombyx mori*	99.34	10.5	100%
R4G844	Putative aminopeptidase of the m17 family	*Rhodnius prolixus*	54.63	4.72	65%
A023F9F8	Putative leucine aminopeptidase 1	*Triatoma infestans*	54.63	4.72	85%
**Proteases (Salivary Gland)**					
R4G8R7	Putative metallopeptidase	*Rhodnius prolixus*	43.05	13.2	100%
R4WJM5	Methionine aminopeptidase 2	*Riptortus pedestris*	38.6	20.6	100%
AFK85026	Aminopeptidase N-10	*Bombyx mori*	99.34	21	100%
AEM97987	Signal peptidase complex subunit SPC25	*Dipetalogaster maximus*	22.05	26.9	100%
A9CPH3	26,29 kDa proteinase-like cysteine protease	*Plautia stali*	58.75	9.7	100%
AEO94539	Aspartate protease cathepsin D	*Triatoma infestans*	42.66	16.2	100%
XP_969056	Similar to ubiquitin specific peptidase 14	*Tribolium castaneum*	53.95	8.6	100%
XP_971897	Similar to metalloprotease	*Tribolium castaneum*	118	15.7	100%
**Nucleases (Salivary Gland)**					
XP_001601829	Exosome complex exonuclease RRP44-like	*Nasonia vitripennis*	112	12.3	100%

The mass spectrometry data for proteases and nucleases listed in Table 1 are provided in Supplementary File 2.
